# Src Homology Phosphatase 2 Disruption in Podocytes Attenuates Markers of Aristolochic Acid 1-Induced Renal Injury, Inflammation, and Fibrosis in Mice

**DOI:** 10.3390/ijms27209334

**Published:** 2026-10-21

**Authors:** Ming-Fo Hsu, Fawaz G. Haj

**Affiliations:** 1 Department of Nutrition, University of California Davis, Davis, CA 95616, USA; mfhsu@ucdavis.edu; 2 Comprehensive Cancer Center, University of California Davis, Sacramento, CA 95817, USA; 3 Division of Endocrinology, Diabetes, and Metabolism, Department of Internal Medicine, University of California Davis, Sacramento, CA 95817, USA

**Keywords:** aristolochic acid, aristolochic acid nephropathy, Src homology phosphatase 2, podocyte, renal fibrosis, inflammation

## Abstract

Aristolochic acid nephropathy (AAN) is a tubulointerstitial kidney disease caused by ingestion of aristolochic acids (AA). It is a global health concern, with no established disease-modifying therapy. Src homology phosphatase 2 (Shp2; encoded by *Ptpn11*) is a ubiquitously expressed protein tyrosine phosphatase that is implicated in renal development and function, but its contribution to AAN is unknown. Mice with podocyte-specific Shp2 disruption (*Ptpn11^fl/fl^*;Pod-Cre; KO) and controls (*Ptpn11^fl/fl^*) were subjected to weekly intraperitoneal injections of saline or aristolochic acid 1 (AA1, 5 mg/kg) for 4 consecutive weeks. Afterward, animals were sacrificed at 6 and 11 weeks. Renal Shp2 protein and immunofluorescence staining, including in glomeruli, were elevated following AA1 challenge. Relative to controls, KO mice exhibited attenuation of AA1-evoked changes in renal function indices, including kidney-to-body weight ratio, blood urea nitrogen, and urine albumin-to-creatinine ratio. Additionally, AA1-induced elevation in blood pressure was reduced in KO mice. At the molecular level, Shp2 deficiency was associated with significantly lower AA1-induced renal expression of inflammatory (*Ccl2*, *Tnf*) and fibrotic (*Acta2*, *Col1a1*) markers compared with controls. Histologically, AA1-induced renal interstitial collagen deposition, evaluated by picrosirius red staining, was significantly reduced in KO mice compared with controls. These findings indicate that podocyte Shp2 deficiency confers beneficial effects and attenuates AA1-induced renal injury, inflammation, and fibrosis, and identify Shp2 as a target in AAN.

## 1. Introduction

Aristolochic acid nephropathy (AAN) is a tubulointerstitial kidney disease that is caused by the ingestion of aristolochic acids (AAs), compounds present primarily in Aristolochia and some Asarum plant species [1]. The disorder is characterized by tubular atrophy, acute proximal tubule necrosis, and interstitial fibrosis, with progression to chronic kidney disease (CKD) and end-stage renal disease (ESRD) [2,3]. Strikingly, most patients progress to ESRD within a few years of diagnosis [4]. Additionally, AAN is strongly associated with upper tract urothelial cancer (UTUC) [5,6], and herbal remedies containing Aristolochia were classified as Group 1 human carcinogens (with aristolochic acid subsequently classified as Group 1) [7]. AAN was first reported in 1993 in a cohort of young Belgian females who consumed weight-loss herbs containing AA [8,9]. Subsequent molecular-epidemiologic studies established that the Balkan endemic nephropathy (BEN) is due to the consumption of flour contaminated with AA [10]. Moreover, in regions where AA-containing traditional herbal medicines are used, there is a high risk of AAN [11,12,13]. Accordingly, human exposure to AA remains a global health concern, and its incidence is likely higher than initially recognized [14]. To date, no disease-modifying therapy for AAN has been established, and management remains largely supportive, with exposure prevention as the most effective strategy.

Mechanistically, AAN pathogenesis is an interplay of multiple injurious factors, including selective renal uptake, DNA damage and mutagenesis, oxidative stress, inflammation, and fibrosis [15]. The primary cellular target of AA is the proximal tubular epithelial cells (PTECs), where organic anion transporters 1 and 3 (OAT1 and OAT3) mediate the uptake contributing to cytotoxicity and inflammation [16,17]. AAs are activated to reactive intermediates that form long-lasting AA-derived DNA adducts, which serve as biomarkers of exposure and exhibit a characteristic mutational signature featuring A:T → T:A transversions [18,19]. Notably, AA exposure increases reactive oxygen species (ROS), disrupts mitochondrial function, and triggers apoptosis [20,21,22]. Additionally, AA induces inflammatory responses by activating Toll-like receptors and recruiting cytotoxic T cells, which contribute to renal injury [17,23]. Moreover, chronic or repeated injury by exposure to AA induces epithelial–mesenchymal-like changes in proximal tubular cells with the accumulation of vimentin and α-smooth muscle actin along with overexpression of transforming growth factor-β (TGF-β), culminating in progressive tubular atrophy and interstitial fibrosis, hallmark features of chronic AAN [20,24,25]. Furthermore, in addition to PTECs, AA causes significant glomerular podocyte damage, leading to mitochondrial dysfunction and contributing to foot-process effacement and albuminuria [26].

The Src homology phosphatase 2 (Shp2; encoded by *Ptpn11*) is a ubiquitously expressed protein tyrosine phosphatase (PTP) implicated in renal function. During kidney development, Shp2 modulates branching morphogenesis, and its disruption causes significant defects in developing mouse kidneys [27]. In the mature kidney, Shp2 is expressed in endothelial cells, tubular epithelial cells, mesangial cells, and glomerular podocytes, where it regulates glomerular filtration barrier function, growth factor signaling, and inflammatory signaling. We previously showed that podocyte-specific Shp2 disruption mitigates lipopolysaccharide-induced renal injury [28] and confers renoprotective effects against hyperglycemia [29]. More recently, we report that Shp2 participates in coordinated signaling networks with other PTPs to modulate a key signaling node, including vimentin, positioning this phosphatase as a central regulator of insulin signaling and cytoskeletal dynamics in podocytes [30]. Notably, genetic or pharmacological targeting of Shp2 ameliorates acute renal injury, diabetic kidney disease, and lupus nephritis [31,32,33]. Collectively, these findings establish a significant role for Shp2 in renal function, but its potential contributions to AAN remain to be determined.

## 2. Results

### 2.1. Elevated Renal Shp2 Expression in Mice with AA1 Challenge

To investigate Shp2 regulation in AAN, we evaluated renal Shp2 expression in a relevant AAN mouse model, as detailed in Methods. The podocyte-specific disruption of Shp2 in this line (*Ptpn11^fl/fl^*;Pod-Cre) was established and validated previously using complementary approaches [28], and subsequently used in other studies [29,30]. Briefly, control (Ctrl; *Ptpn11^fl/fl^*) and podocyte-specific Shp2 knockout (KO; *Ptpn11^fl/fl^*;Pod-Cre) male mice were subjected to saline and AA1 (5 mg/kg, once per week) intraperitoneal injections for 4 consecutive weeks, followed by remodeling periods of 2 and 7 weeks for renal damage development (Figure 1A). Animals were sacrificed at 6 and 11 weeks, and blood and kidney samples were collected for further analyses. Immunoblots of whole kidney lysates demonstrate significantly elevated Shp2 protein expression at 6 and 11 weeks, with the highest expression at the former (Figure 1B). Similarly, immunofluorescence staining of Shp2 in the kidney indicated elevated staining at 6 and 11 weeks, including the glomeruli (Figure 1C). Because podocytes constitute only a small fraction of the kidney, the whole-kidney immunoblots and whole-section immunostaining reflect total renal Shp2 and were performed to assess AA1-induced regulation of renal Shp2. Together, these findings indicate elevated renal Shp2 expression in a mouse model of AAN and suggest that this upregulation may contribute to disease pathogenesis.

### 2.2. Mice with Podocyte Shp2 Disruption Partially Ameliorate the Adverse Effects of AA1 Challenge on Renal Functions

To determine Shp2’s potential role in AAN pathogenesis, we examined the effects of podocyte Shp2 deficiency on several renal and physiological indices under basal and AA1 challenge. Indeed, AA1 treatment significantly reduced body weight (Figure 2A) and kidney weight (Figure 2B) in both genotypes at 6 and 11 weeks. Notably, the AA1-induced reduction in the kidney-to-body weight ratio was partially mitigated in KO animals compared with controls at 11 weeks (Figure 2C). Next, we measured blood urea nitrogen (BUN) and urine albumin-to-creatinine ratio (ACR) to assess renal function. Of note, AA1 significantly increased BUN levels at 6 and 11 weeks, but the induction was substantially lower in the KO animals compared with controls at 11 weeks (Figure 2D). Additionally, AA1-caused elevation of ACR level was partially but significantly reduced in the KO mice at 1 week after AA1 administration (Figure 2E). Moreover, we determined blood pressure at 5 and 10 weeks to further evaluate the impact of Shp2 deficiency on AA1-induced renal injury and secondary hypertension. Remarkably, the AA1-evoked increase in systolic pressure (Figure 2F) and diastolic pressure (Figure 2G) was significantly attenuated in mice with Shp2 deficiency compared to the control animals. Collectively, these results demonstrate that podocyte Shp2 disruption partially mitigates AA1-induced toxicity and renal dysfunction in mice.

### 2.3. Podocyte Shp2 Deficiency Is Associated with Reduced Markers of Renal Inflammation and Fibrosis in AA1-Treated Animals

To investigate the molecular correlates underlying the beneficial effects of Shp2 deficiency under AA1 challenge, we evaluated alterations in renal inflammation and fibrosis markers. AA1-induced gene expression of the inflammatory markers monocyte chemoattractant protein-1 (*Ccl2*/MCP-1) and tumor necrosis factor (*Tnf*) was partially but significantly lower in the kidneys of Shp2-deficient mice at 6 and 11 weeks (Figure 3A). Additionally, renal fibrosis markers were examined by the gene expression of actin alpha 2, smooth muscle (*Acta2*) and collagen type I alpha 1 chain (*Col1a1*). Notably, KO mice exhibited significantly lower *Acta2* and *Col1a1* levels with AA1 treatment (at 11 and 6 weeks, respectively), compared with control animals (Figure 3B). To corroborate the molecular and functional findings with direct histological evidence of renal injury, we performed picrosirius red (PSR) staining to assess interstitial collagen deposition in control and KO mice (n = 3/group). In saline-treated animals of both genotypes, the cortical PSR-positive area was minimal (Figure 3C,D). AA1 challenge markedly increased interstitial collagen deposition in both genotypes at 6 and 11 weeks. Notably, the AA1-evoked increase in PSR-positive area was significantly lower in KO than in Ctrl mice at 11 weeks, with a comparable trend at 6 weeks (Figure 3D). These histological findings align with the reduced renal *Col1a1* and *Acta2* expression (Figure 3B), indicating that podocyte Shp2 deficiency mitigates AA1-induced interstitial fibrosis. Together, these observations suggest a potential role for Shp2 in modulating AA1-induced renal inflammation and fibrosis.

## 3. Discussion

Shp2 expression and enzymatic activity are upregulated in several kidney disorders. We demonstrate elevated renal Shp2 expression in mouse models of LPS- and hyperglycemia-induced kidney injury [28,29]. Additionally, Shp2 activity in the kidney, assessed by phosphorylation, a commonly used surrogate for its activation, is increased in a model of acute injury induced by sequential exposure to hemorrhagic shock and sepsis [31]. Similarly, in some human glomerulopathies, Shp2 phosphorylation, which correlates with its activity, is increased [34]. Moreover, Shp2 activity is elevated in patients with diabetic nephropathy and db/db mice [32]. These findings that Shp2 expression is upregulated in a mouse model of AAN align with these reports. However, the factor(s) modulating Shp2 upregulation in AAN remain undetermined.

Although the contribution of elevated Shp2 to AAN remains unresolved, we demonstrate that podocyte deficiency of this phosphatase manifests partial but significant protection in an experimental model of the disease. The amelioration of AA1-induced elevations in BUN and ACR is consistent with the beneficial renal effects of genetic and pharmacological inactivation of this phosphatase in other renal injury models [28,29,31,32,33,35]. Additionally, Shp2 deficiency partially attenuates AA1-triggered elevation in blood pressure. Although hypertension is not a prominent early feature of AAN, in BEN, later-onset hypertension has been documented [36]. Recent evidence suggests that AA1 induces mitochondrial dysfunction in the high-energy-consuming heart and kidneys, which may contribute to later, milder hypertension in AAN [37]. A limitation is that the blood pressure was measured by non-invasive tail-cuff rather than radiotelemetry; although this method was applied identically to both genotypes, it does not capture the 24-h or diastolic blood pressure dynamics. While the mechanisms underlying Shp2 regulation of blood pressure in AAN remain to be established, it is reasonable to speculate that they include several interconnected processes. Conceivably, podocyte-specific Shp2 deletion may confer renoprotection in AAN by influencing other renal cells, including the PTECs and interstitial cells, through several paracrine mechanisms. Podocytes function as a hub for intercellular communication and secrete extracellular vesicles (EVs) and factors that impact tubular cell function [38,39]. Of note, Shp2 regulates the biogenesis of small EVs through dephosphorylation of Syntenin (Tyr46) [40], suggesting that the podocyte deficiency of this phosphatase could alter EV-mediated cell–cell communication. Supporting this crosstalk model, a podocyte-restricted deficiency nonetheless decreased tubulointerstitial collagen deposition, in keeping with podocyte-to-tubule and EV-mediated signaling. Additionally, podocyte Shp2 deficiency ameliorates the renal inflammatory profile following challenges [28,29] that would otherwise contribute to tubular inflammation. Thus, multiple mechanisms may account for the mitigation of glomerular injury and tubular damage in AAN by targeting this phosphatase in podocytes.

At the molecular level, the current findings suggest that Shp2 signaling intersects with pathways implicated in AAN pathogenesis, including but not limited to inflammation and fibrosis. Inflammation is a significant contributor to AAN pathogenesis. Macrophage infiltration is an early feature of the disease in humans and experimental models. Notably, targeting macrophages with fms-I (a selective inhibitor of the macrophage colony-stimulating factor receptor, c-fms/CSF1R) in preventive or therapeutic settings ameliorates renal inflammation, including by inhibiting NF-κB signaling, and improves renal dysfunction [41]. Additionally, targeting the C3a/C3aR axis in macrophages may represent a therapeutic approach for treating renal fibrosis in AAN [42]. Moreover, inhibition of TNF-α (using etanercept) decreases markers of inflammation and partially, but significantly, attenuates albuminuria and renal fibrosis in AAN [43]. Indeed, pharmacological inhibition of Shp2 attenuates renal inflammation via the ERK/NF-κB pathway in diabetic nephropathy and lupus nephritis [32,44]. Fibrosis is a prominent feature of AAN, characterized by progressive interstitial fibrosis, myofibroblast activation, and matrix accumulation [3]. TGF-β is an established signaling axis that modulates this process and promotes fibroblast-to-myofibroblast differentiation [3,45]. The observed mitigation of AA1-evoked *Acta2* and *Col1a1* expression upon Shp2 deficiency likely constitutes a mechanistic link bridging Shp2 signaling and fibrosis in AAN. Supporting this, the attenuation of AA1-induced PSR-positive interstitial collagen in Shp2-deficient kidneys provides direct histological confirmation of reduced fibrosis, consistent with lower Acta2 and Col1a1 expression and preserved renal function. Indeed, Shp2 can promote TGF-β-evoked JAK2/STAT3 signaling that drives fibrosis and conversely, inactivation of Shp2 mitigates fibrosis [46]. However, we cannot rule out additional/alternative Shp2-regulated mechanism(s). For example, vimentin accumulation is a hallmark of EMT in AAN [3]. We recently identified vimentin as a Shp2 substrate in the kidney [30], suggesting that this phosphatase may modulate vimentin-dependent AA1 injury outcomes. Collectively, these findings uncover a potential role for Shp2 in AA1-induced injury and suggest that therapeutic targeting of Shp2 may provide a novel strategy to ameliorate renal dysfunction in AAN. Accordingly, Shp2 pharmacological inhibitors currently in clinical trials in humans for other diseases [47] are viable agents to evaluate for AAN treatment.

## 4. Materials and Methods

### 4.1. Reagents

Antibodies for Shp2 (sc-280) and beta-Actin (sc-47778) were from Santa Cruz Biotechnology (Dallas, TX, USA). Aristolochic acid 1 (AA1) sodium salt was purchased from Sigma-Aldrich (A9451, St. Louis, MO, USA). Fast Green powder (A16520.06) and Picro-Sirius Red Stain Kit (PSR-1) were obtained from Thermo Fisher Scientific (Waltham, MA, USA) and ScyTek Laboratories (Logan, UT, USA), respectively.

### 4.2. Animal Study

The mice with podocyte-specific disruption of Shp2 (*Ptpn11^fl/fl^*;Pod-Cre) were generated by crossing Shp2 floxed (*Ptpn11^fl/fl^*) mice with podocin-Cre transgenic mice (Jackson Laboratories, Bar Harbor, ME, USA) as described previously [28]. All animals (2 mice/cage) were maintained on a 12-h light/dark cycle in a temperature-controlled facility, with free access to water and food (standard laboratory chow diet). For the study, 10- to 12-week-old control (*Ptpn11^fl/fl^*) and podocyte Shp2 knockout (*Ptpn11^fl/fl^*;Pod-Cre) male mice (40 in total) were administered saline (vehicle control group; n = 8/group) and AA1 (5 mg/kg body weight; n = 12/group) by intraperitoneal injection once a week for a continuous 4 weeks. AA1 was prepared in saline (0.9% sodium chloride/water, *w*/*v*), and the dosing volume of saline and AA1 was 10 mL/kg body weight. Saline groups were sacrificed at 11 weeks, and AA1-dosed mice were sacrificed at 6 (n = 6/group) and 11 (n = 6/group) weeks after the first injection. Rapid urine collection was achieved by placing a mouse on a clean plastic wrap to induce urination. At the end of treatment, the submandibular bleeding method (5 mm Goldenrod sterile single-use animal lancet without anesthesia) was applied to collect blood samples in EDTA tubes. The plasma fraction was isolated by centrifugation at 2000× *g* for 15 min at 4 °C. Systolic and diastolic blood pressure were measured at 5 and 10 weeks after the first injection using a non-invasive CODA high-throughput tail-cuff system (Kent Scientific Corporation, Torrington, CT, USA), which employs volume-pressure recording (VPR). Unlike older flow-based cuff instruments, VPR has been validated against simultaneous radiotelemetry and provides accurate measurements across the physiological blood pressure range in mice [48,49]. Measurements were obtained during the light phase by a single trained operator using a standardized protocol applied identically to both genotypes. Final euthanasia was performed by cervical dislocation, and tissue samples were collected for further experiments. Plasma albumin, creatinine, and blood urea nitrogen concentrations were determined by the manufacturer’s instructions from Sigma-Aldrich (MAK124, MAK080, MAK006). The Institutional Animal Care and Use Committee at the University of California, Davis approved all mouse studies (animal protocol #24716; 18 December 2025).

### 4.3. Immunoblotting Analysis

The dissected kidney was snap-frozen in liquid nitrogen and stored at −80 °C. Frozen tissue was ground to a fine powder using a mortar and pestle with liquid nitrogen. Total lysate was extracted using radioimmunoprecipitation assay buffer containing 10 mM Tris-HCl (pH 7.4), 150 mM NaCl, 0.1% SDS, 1% Triton X-100, 1% sodium deoxycholate, 5 mM EDTA, 1 mM NaF, 1 mM sodium orthovanadate, and protease inhibitors. The protein fraction was isolated by centrifugation at 15,000× *g* for 10 min at 4 °C, and concentration was determined using a BCA Protein Assay Kit (Thermo Fisher Scientific). Proteins were resolved by SDS-PAGE and transferred to a PVDF membrane. Primary antibodies (Shp2, 1:5000; Actin, 1:10,000) were incubated at 4 °C overnight, and HRP-conjugated secondary antibodies were applied to the membrane for 60 min at room temperature. Protein bands were visualized with HyGLO Chemiluminescent solution (Denville Scientific Inc., Swedesboro, NJ, USA) and HyBlot CL autoradiography film, and quantified using a FluorChem 9900 (Alpha Innotech, Minneapolis, MN, USA).

### 4.4. Histology

4% paraformaldehyde-fixed mouse kidney tissues were embedded and sectioned by the UC Davis Anatomic Pathology Service. Samples were deparaffinized and rehydrated in xylene, ethanol, and ethanol/PBS solutions. For immunofluorescence staining, we performed heat-mediated antigen retrieval in citrate buffer (10 mM sodium citrate, pH 6.0). Slides were blocked with 3% BSA at room temperature for 60 min, then incubated with Shp2 antibodies (1:200) at 4 °C overnight, followed by Alexa Fluor 488-conjugated secondary antibodies (Thermo Fisher Scientific) for 60 min at room temperature. Stained images were acquired on an Olympus FV1000 confocal microscope (Hachioji, Tokyo, Japan). For PSR staining, deparaffinized and rehydrated kidney sections were incubated with Fast Green solution (10% *w*/*v* in distilled water) for 15 min at room temperature. Slides were washed with a 0.5% acetic acid solution (acidified water) several times (5 min each) to remove excess stain. The samples were then incubated with PSR solution at room temperature for 60 min. Sections were washed several times (5 min each) in the acidified water until the wash solution became colorless. After dehydration through graded ethanols (70%, 95%, 100%) and xylene (twice for 1 min each), the samples were mounted in Permount medium. Images were captured on an Olympus BX51 microscope and analyzed using Fiji (ImageJ version 1.54p; National Institutes of Health, Bethesda, MD, USA).

### 4.5. Real-Time PCR

Kidney samples were homogenized in pre-filled bead mill tubes (2 mL tube with 1.4 mm ceramic beads, Thermo Fisher Scientific) and TRIzol reagent (Invitrogen, Waltham, MA, USA). Total RNA was purified using a Direct-zol RNA Miniprep Plus Kit (Zymo Research, Irvine, CA, USA), and its quantity and quality were measured with a NanoDrop One (Thermo Fisher Scientific). Then cDNA was synthesized using the High-Capacity cDNA Reverse Transcription Kit (Applied Biosystems, Waltham, MA, USA), and samples were mixed with SsoAdvanced Universal SYBR Green Supermix (Bio-Rad, Hercules, CA, USA) for the PCR reaction. Target gene expression was normalized to TATA box-binding protein (*Tbp*), and the relative fold change was calculated using the ∆∆CT method on an arbitrary scale. Primer pairs used in the study are: *Ccl2*, forward-CCCAATGAGTAGGCTGGAGA, reverse-TCTGGACCCATTCCTTCTTG; *Tnf*, forward-TGACGTGGAACTGGCAGAAGAG, reverse-TTGCCACAAGCAGGAATGAGA; *Acta2*, forward-TCAGCGCCTCCAGTTCCT, reverse-TAAAAAAACCACGAGTAACAA; *Col1a1*, forward-AGAGGCGAAGGCAACAGTCG, reverse-GCAGGGCCAATGTCTAGT; *Tbp*, forward-TGAGAGCTCTGGAATTGTAC, reverse-CTTATTCTCATGATGACTGCAG.

### 4.6. Statistical Analyses

Quantification data are expressed as mean ± standard error of the mean and summarized in a bar graph, with individual data points shown. Statistical analyses were performed using Excel with a Real Statistics (Release 9.9) plug-in (www.real-statistics.com, accessed on 12 October 2026). Differences at *p* < 0.05 were considered significant by one-way ANOVA with post hoc Tukey’s test (Figure 1B) and pairwise *t*-test (Figure 2 and Figure 3) for multi-group comparison.

## Figures and Tables

**Figure 1:**
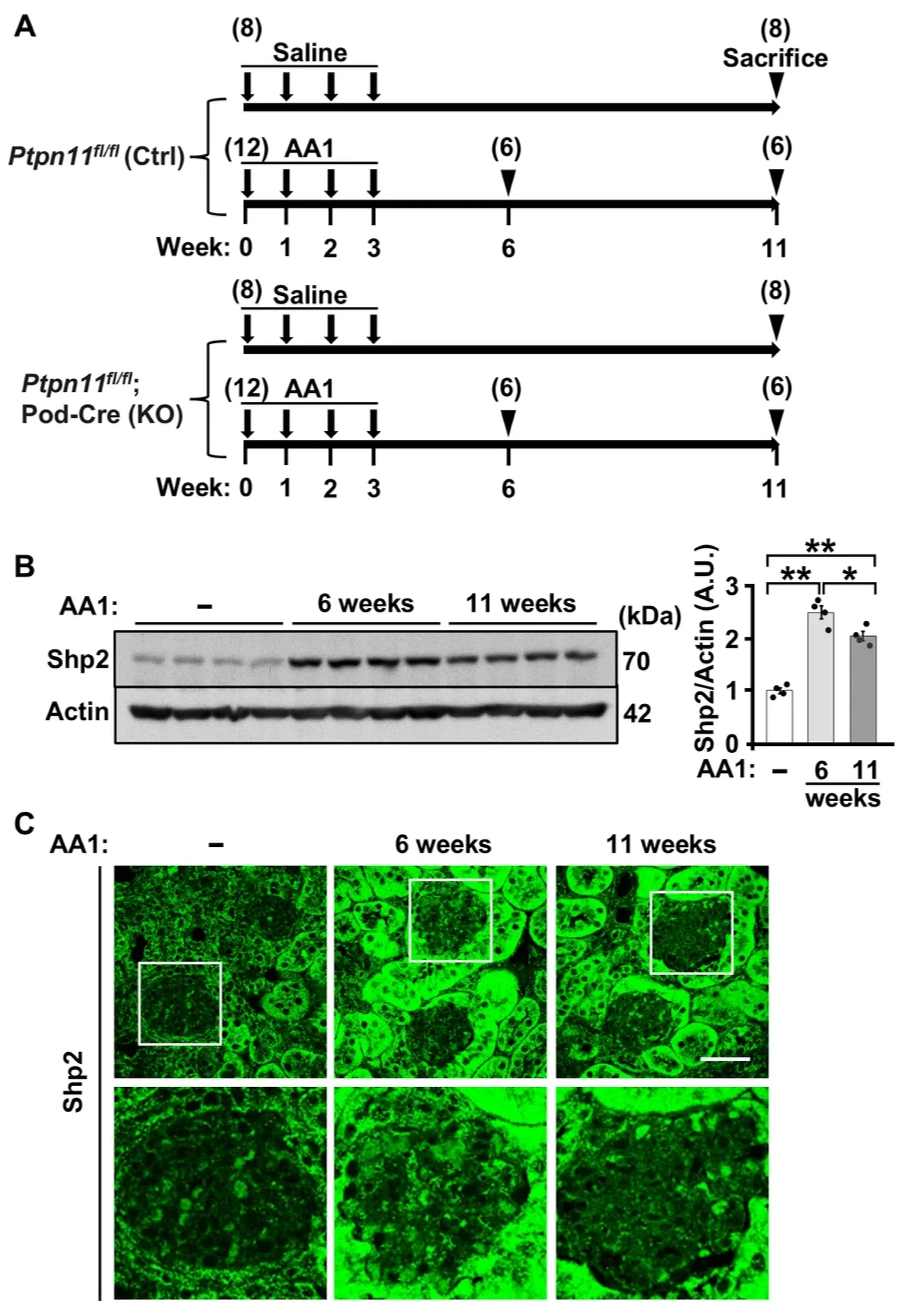
Elevated renal Shp2 expression in mice with chronic AA1 treatment. (**A**) Schematic of experimental timeline for AA1 administration (5 mg/kg body weight, arrows) and mouse sacrifice (6 and 11 weeks, arrowheads). (**B**) Immunoblots of Shp2 and Actin in renal lysates (*n* = 4/group), and (**C**) immunostaining of Shp2 in renal sections (*n* = 3/group) from mice without (−, sacrificed at 11 weeks) and with AA1 (sacrificed at 6 and 11 weeks). Shp2 protein levels in (**B**) were normalized to Actin, and the ratios were summarized in a bar plot, with dots representing individual animals. * *p*  <  0.05 and ** *p*  <  0.01. A.U., arbitrary units. Glomeruli in (**C**) were enlarged from white boxes. Scale bar: 50 μm.

**Figure 2:**
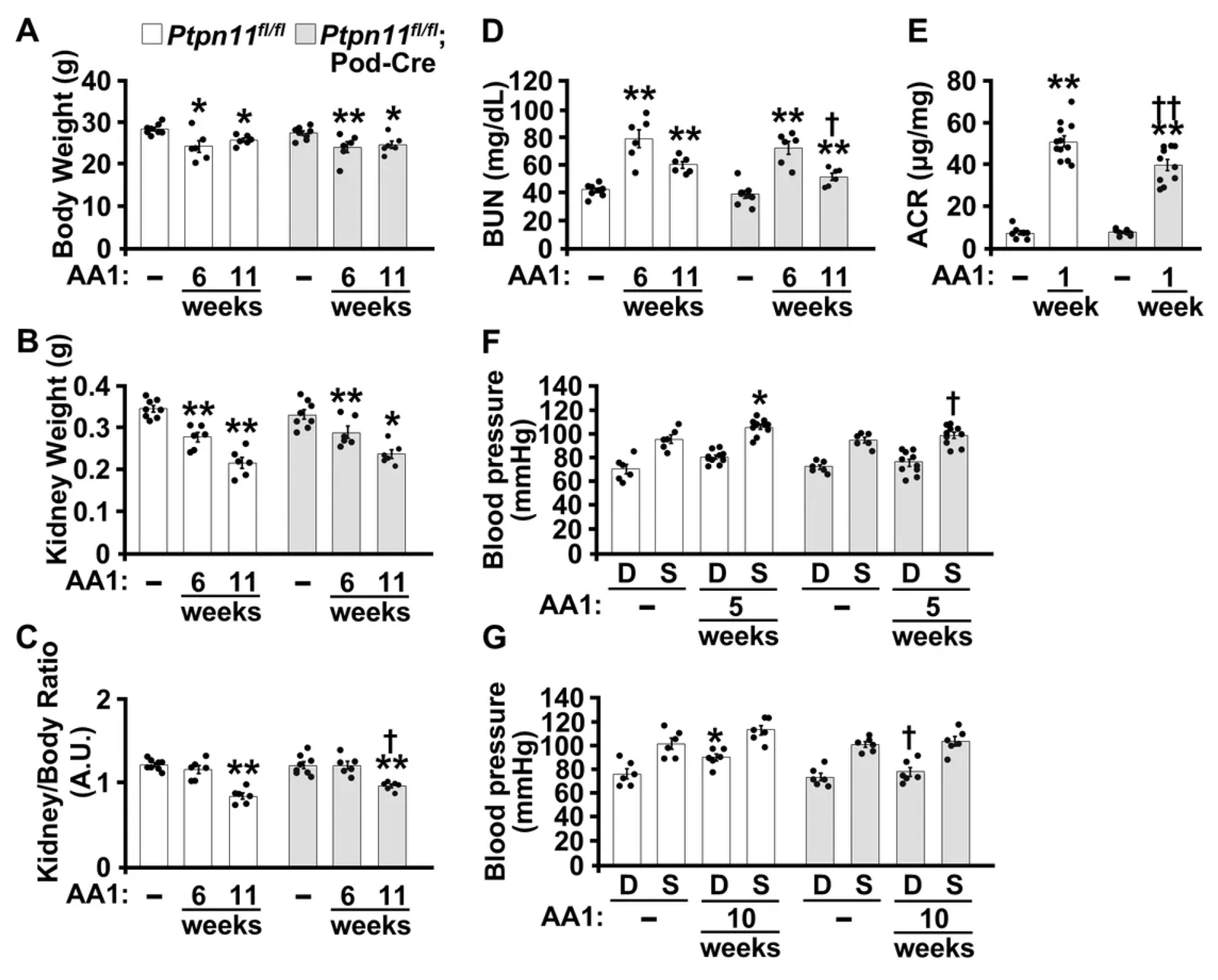
Mice with podocyte Shp2 disruption are more resistant to AA1-induced renal injury. (**A**) Body weight, (**B**) kidney weight, (**C**) kidney/body weight ratio, and (**D**) blood urea nitrogen (BUN) of control (*Ptpn11^fl/fl^*) and podocyte Shp2 knockout (*Ptpn11^fl/fl^*;Pod-Cre) mice (*n* = 6~8/group) without (−, sacrificed at 11 weeks) and with AA1 (sacrificed at 6 and 11 weeks). (**E**) Albumin–creatinine ratio (ACR) in urine samples (*n* = 8~11/group) from mice without and with AA1 at 1 week. Diastolic (D) and systolic (S) blood pressure in mice (*n* = 6~10/group) without and with AA1 at (**F**) 5 weeks and (**G**) 10 weeks. Data were summarized in bar plots with dots representing individual animals. * *p*  <  0.05 and ** *p*  <  0.01, without and with AA1 in mice with the same genotype. ^†^ *p*  <  0.05 and ^††^ *p*  <  0.01, *Ptpn11^fl/fl^* and *Ptpn11^fl/fl^*;Pod-Cre mice with the same treatment. A.U., arbitrary units.

**Figure 3:**
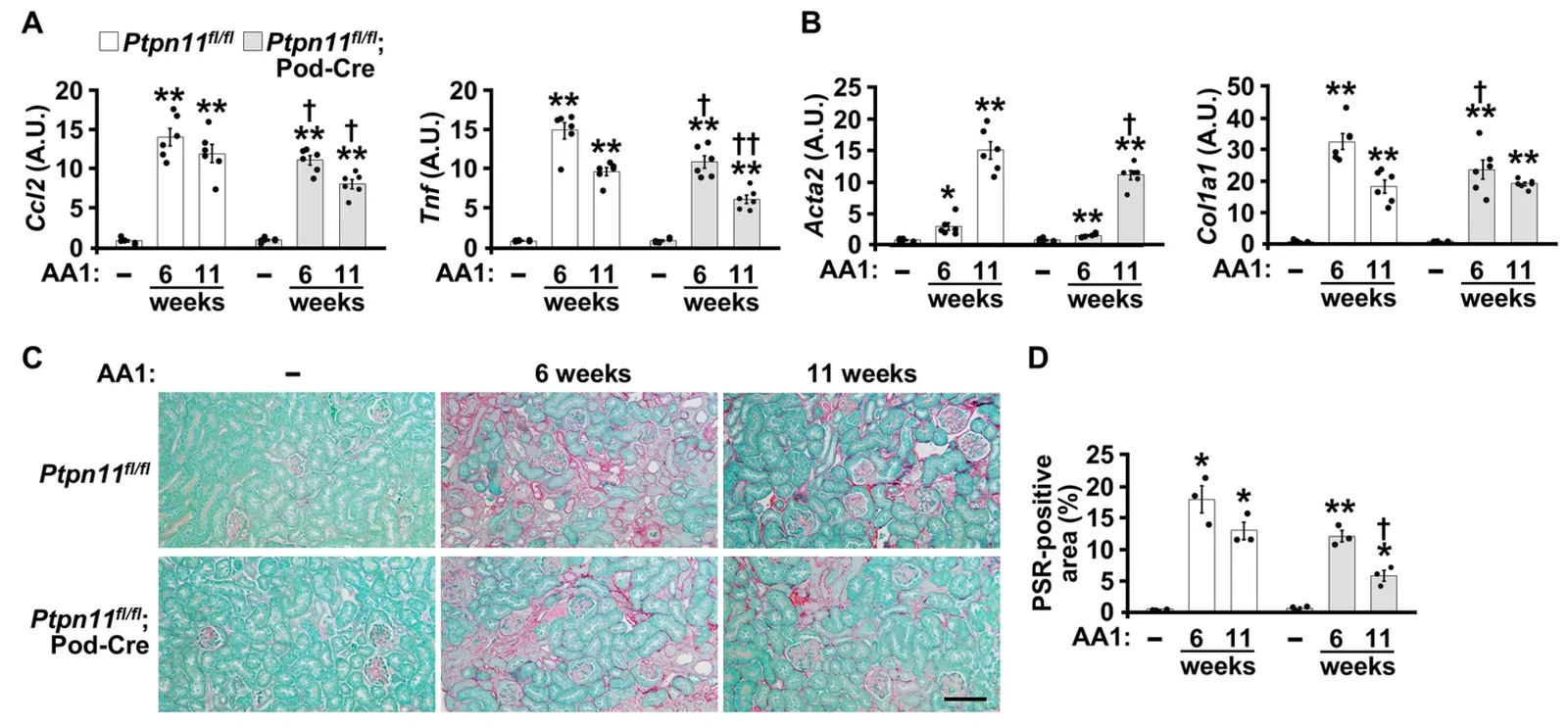
Attenuated AA1-induced inflammation and fibrosis in mice with podocyte Shp2 disruption. Gene expression (*n* = 6/group) of (**A**) Ccl2, Tnf, and (**B**) Acta2, Col1a1 in renal samples, and (**C**) representative picrosirius red (PSR)-stained cortical kidney sections from control (*Ptpn11^fl/fl^*) and podocyte Shp2 knockout (*Ptpn11^fl/fl^*;Pod-Cre) mice without (−, sacrificed at 11 weeks) and with AA1 (sacrificed at 6 and 11 weeks). Collagen was stained red-pink against a Fast Green counterstain, and the PSR-positive area (*n* = 3/group) was quantified in (**D**). Data were summarized in a bar plot with dots representing individual animals. * *p*  <  0.05, ** *p*  <  0.01, without and with AA1 in mice with the same genotype, and ^†^ *p*  <  0.05, ^††^ *p*  <  0.01, *Ptpn11^fl/fl^* and *Ptpn11^fl/fl^*;Pod-Cre mice with the same treatment. A.U., arbitrary units. Scale bar: 100 μm.

## Data Availability

The original contributions presented in this study are included in the article.
